# Tripartite ATP-independent Periplasmic (TRAP) Transporters Use an Arginine-mediated Selectivity Filter for High Affinity Substrate Binding*

**DOI:** 10.1074/jbc.M115.656603

**Published:** 2015-09-05

**Authors:** Marcus Fischer, Adam P. Hopkins, Emmanuele Severi, Judith Hawkhead, Daniel Bawdon, Andrew G. Watts, Roderick E. Hubbard, Gavin H. Thomas

**Affiliations:** From the York Structural Biology Laboratory, Departments of ‡ Chemistry and; § Biology (Area 10), University of York, P. O. Box 373, York YO10 5YW and; the ¶ Department of Pharmacy and Pharmacology, University of Bath, Claverton Down, Bath BA2 7AY, United Kingdom

**Keywords:** bacterial pathogenesis, membrane transporter reconstitution, protein structure, sialic acid, transporter

## Abstract

Tripartite ATP-independent periplasmic (TRAP) transporters are secondary transporters that have evolved an obligate dependence on a substrate-binding protein (SBP) to confer unidirectional transport. Different members of the DctP family of TRAP SBPs have binding sites that recognize a diverse range of organic acid ligands but appear to only share a common electrostatic interaction between a conserved arginine and a carboxylate group in the ligand. We investigated the significance of this interaction using the sialic acid-specific SBP, SiaP, from the *Haemophilus influenzae* virulence-related SiaPQM TRAP transporter. Using *in vitro*, *in vivo,* and structural methods applied to SiaP, we demonstrate that the coordination of the acidic ligand moiety of sialic acid by the conserved arginine (Arg-147) is essential for the function of the transporter as a high affinity scavenging system. However, at high substrate concentrations, the transporter can function in the absence of Arg-147 suggesting that this bi-molecular interaction is not involved in further stages of the transport cycle. As well as being required for high affinity binding, we also demonstrate that the Arg-147 is a strong selectivity filter for carboxylate-containing substrates in TRAP transporters by engineering the SBP to recognize a non-carboxylate-containing substrate, sialylamide, through water-mediated interactions. Together, these data provide biochemical and structural support that TRAP transporters function predominantly as high affinity transporters for carboxylate-containing substrates.

## Teaser

**Background:** *Haemophilus influenzae* requires a substrate-binding protein (SBP)-dependent TRAP transporter to acquire sialic acid.

**Results:** A conserved arginine residue in the SBP is essential for the high affinity and carboxylate specificity of the TRAP transporter.

**Conclusion:** The arginine/carboxylate interaction in TRAP SBPs restricts substrate range to carboxylate-containing substrates.

**Significance:** The study reveals the mechanism by which a key bimolecular interaction underpins bacterial virulence.

## Introduction

Secondary transporters use preformed electrochemical gradients to energize the concentrative movement of chemicals across biological membranes and are ubiquitous in living cells. Bacteria use secondary transporters primarily for uptake of nutrients from their environment, and often bacterial genomes can encode over 100 different secondary transporters (1), which will likely transport a very wide variety of substrates ranging from sugars, amino acids, nucleotides, fatty acids, inorganic ions, and organic acids (1, 2). Many bacteria and archaea, especially those that live in the sea and other marine environments (7), and also a range of human pathogens, use a particular family of secondary transporters called tripartite ATP-independent periplasmic (TRAP)^6^ transporters that also have a substrate-binding protein (SBP) component (3–5). The SBP is either free in the periplasm of Gram-negative bacteria or anchored to the cell membrane in Gram-positive bacteria and is a transporter component traditionally found in prokaryotic ATP-binding cassette uptake systems (6, 7). Through the characterization of the SiaPQM sialic acid-specific TRAP transporter from *Haemophilus influenzae*, which is essential for host colonization, some of the properties conferred by using the SBP have been elucidated at the biochemical level (8–11). TRAP transporters have three subunits, the SBP and two membrane proteins of unequal size. The M subunit is a 12-transmembrane helix (TMH)-containing protein that is a member of the ion transporter superfamily and likely forms the translocation channel, whereas the Q subunit is a 4-TMH protein of essential but undefined function. In the *H. influenzae* SiaPQM TRAP transporter, the SBP is SiaP, and the two membrane proteins that constitute the TRAP transporter, SiaM and SiaQ, are naturally fused into a single predicted 17-TMH protein, SiaQM. The SBP initiates the transport process by binding the substrate. By being located on the extra-cytoplasmic side of the membrane, it imposes directionality on a secondary transporter by delivering the ligand to the SiaQM membrane domains for subsequent translocation into the cell (12).

The first structure of a TRAP SBP (5) revealed that the charge of the carboxylate group of the ligand, sialic acid, was neutralized by an arginine residue, Arg-147, in SiaP, which sequence analysis had revealed to be the most highly conserved residue, being present in 98% of TRAP SBPs of the common DctP family (5). On this basis, the Arg/carboxylate interaction was proposed to be a defining feature of the DctP-TRAP SBPs that might have additional functions in the overall transporter mechanism than simply this electrostatic interaction with the ligand (4), such as triggering of domain closure in the protein. Additional structures of DctP-type SBPs from TRAP transporters that recognize (hydroxy-)ectoine, pyroglutamate, and monocarboxylate keto acids as substrates all have a similarly functioning arginine residue (13–17). By comparing the substrate-binding sites in these different SBP structures, it is clear that each binding site is highly adapted to its particular carboxylate-containing substrate, with the notable exception of the conserved Arg/carboxylate interaction that is observed between the equivalent residues to Arg-147 of SiaP and the carboxylate group of the different substrates (4). A remarkable recent study has added another 29 unique DctP-TRAP SBP structures to the Protein Data Bank, each with an equivalent arginine (18). These data reinforce the key role of this residue in defining the biological function and substrate range of TRAP transporters, but the structural and biochemical consequences of its disruption have not been investigated to date.

To investigate how SiaP recognizes its sialic acid substrate, Johnston *et al.* (8) examined the biological phenotypes of a number of site-directed mutants of SiaP. Residue Arg-147 was mutated to lysine and to alanine. Using a monoclonal antibody that is unable to recognize sialylated lipooligosaccharide (LOS), they demonstrated a complete lack of complementation of a *siaP* strain of non-typable *H. influenzae* by *siaP* genes that contain the Arg-147 mutations (8). In contrast, other mutations of the binding site resulted in complete or partial complementation, suggesting that they were not important for transport and hence the sialylation phenotype. The basis for the lack of LOS sialylation for Arg-147 mutants was not determined but is consistent with Arg-147 playing an important role in transporter function.

In this study we determined the role of the arginine/carboxylate interaction using SiaP. We demonstrate that it is essential for high affinity binding of substrate and transport under physiological conditions of low substrate concentrations but also that its loss can be tolerated at high ligand concentrations. By disrupting this key interaction in a rational way, we show that we can change the substrate specificity of the SBP from binding a carboxylate to binding an amide. Together these data demonstrate a key role for the Arg-147/carboxylate interaction in TRAP transporter function.

## Experimental Procedures

### 

#### 

##### Bacterial Strains and Culture Conditions

For cloning and transformation, *Escherichia coli* DH5α, BW25113, and MC1061 were grown in LB broth (10 g/liter tryptone, 5 g/liter powdered yeast, 10 g/liter NaCl). Protein production and growth on specific carbon sources used M9 minimal medium salts (19) supplemented with 0.4% d-glucose, Neu5Ac, or other carbon source at the indicated concentrations. Antibiotic selection, where appropriate, used 30 μg/ml chloramphenicol, 100 μg/ml ampicillin, and 50 μg/ml kanamycin.

##### Mutagenesis of the siaP Gene

The wild-type *siaP* gene (HI0146) was amplified by PCR from genomic DNA of *H. influenzae* Rd using the primers *siaPfor* 5′-gcggtacctaaaagaaggagatatacatatgatgaaattgacaaac-3′ and *siaPhis6rev* 5′-ccgctcgagttagtgatggtgg tgatgatgtggattgattgcttcaatttg-3′. The product was digested with NdeI and XhoI and cloned into pBlueScriptII KS, verified by DNA sequencing, and then subcloned using the same sites into pET21b creating pAH16. To mutate the *siaP* gene in pAH16 directly, we used partially overlapping oligonucleotide primers with a silent StuI restriction site in the reverse primer (20); the primers for R147A were 5′-aaacttgctgtgccaaatgcagcaacaaac-3′ (forward) and 5′-ggcacagcaagtttcaggcctttcatatctgc-3′ (reverse); R147K primers were 5′-aacttaaagtgccaaatgcagcaacaaac-3′ (forward) and 5′-ggcactttaagtttcaggcctttcatatctgc-3′ (reverse); and R147E primers were 5′-aaacttgaagtgccaaatgcagcaacaaac-3′ (forward) and 5′-ggcacttcaagtttcaggcctttcatatctgc-3′ (reverse), creating plasmids pAH35 (R147A), pAH36 (R147E), and pAH37 (R147K). For low copy number expression for *in vivo* growth and uptake experiments, we used pWKS30 (21) as the vector. Either wild-type or mutated *siaP* alleles from pAH16 and pAH35-37 were amplified and digested with NdeI and XhoI before being cloned in a three-way ligation with XhoI-*siaQM*-BamHI and NdeI-, BamHI-cut pWKS30, pAH15 (*siaP-*His_6_), pES15 (R147A), pES16 (R147K), and pES17 (R147E). To mutate the *siaP* gene in pAH16 directly, we used partially overlapping oligonucleotide primers with a silent NcoI restriction site in the reverse primer (19); F170Wfor 5′-atggcatggtctgaagtttatcttgcgttac-3′ and F170Wrev 5′-cttcagaccatgccatgggtgttggtgatgc-3′, creating pAH45. The same primers were used to introduce this point mutation in the pAH35-37 series creating pAH66, pAH64, and pAH65, respectively. The clones were verified by DNA sequencing.

##### Expression and Purification of SiaP Proteins

A flask containing 50 ml of M9 minimal medium containing 0.4% d-glucose was inoculated with *E. coli* BL21 (DE3) pLysS pAH16 or other pET21 derivatives and grown at 37 °C overnight with shaking at 180 rpm. This was inoculated into 625 ml of M9 minimal medium containing 0.4% d-glucose to an *A*_650_ 0.1, grown at 25 °C to an *A*_650_ 0.2–0.3, expression induced by the addition of 1 mm IPTG, and incubated overnight at 25 °C. Induced cells were spun at 4000 × *g* for 20 min at 4 °C, resuspended in 25 ml of 5 mm EDTA, 50 mm Tris, 0.5 m sucrose, pH 8.0, and incubated with 12 mg of lysozyme (chicken egg white; Sigma) at 30 °C for 2 h to prepare the periplasmic fraction. The sample was then centrifuged at 17,000 × *g* for 10 min at 4 °C, and the supernatant was dialyzed against 20 mm Tris/HCl, 300 mm NaCl, pH 7.5 (TBS). This was clarified by centrifugation at 17,000 × *g* for 10 min at 4 °C, and imidazole was added to 12 mm. A 1-ml HisTrap HP column (GE Healthcare) was washed with TBS, 12 mm imidazole using a P-1 peristaltic pump (Amersham Biosciences). The dialyzed and clarified periplasmic fraction was loaded onto the column at a 2–5 ml min^−1^, washed with 20 column volumes of TBS, 20 mm imidazole, and the protein eluted with TBS 400 mm imidazole. Protein-containing fractions were visualized using SDS-PAGE, pooled, and concentrated using Vivaspin 2 ultrafiltration spin columns with a 5-kDa molecular mass cutoff (Sartorius).

##### Protein Fluorescence Spectroscopy

Protein fluorescence experiments were performed in a 3-ml quartz cuvette (Starna) using a FluoroMax2 (Instruments SA, Inc.) with an LTD6 water bath (Grant) controlled with the supplied software, DataMax-Std version 2.20. SiaP contains no tryptophans, and so the protein was excited for tyrosine fluorescence. 0.05 μm protein in 50 mm Tris/HCl, pH 8.0, was excited at 281 nm with slit widths of 5–10 nm to give a signal intensity of 2–3 × 10^6^ units. Ligand was added at concentrations and times to produce spectra and time course titrations. For titrations, the cumulative fluorescence change was plotted using SigmaPlot (version 10.0), and the *K_d_* value was determined using a fit to a simple hyperbolic curve. For the F170W/R147K mutant titrations, protein was used at 0.5 μm. To obtain the required millimolar additions of Neu5Ac for titrations, additions of 40 μl of ligand were added, and so a dilution only control was run in parallel to remove dilution effects from the calculation of *K_D_* values.

##### Circular Dichroism

CD spectra were determined using a J-810 spectropolarimeter (Jasco) controlled by the supplied software Spectra Manager version 1.53.00 and maintained at constant temperature by the Peltier unit PFD-425S. The spectrum of 0.1 mg/ml protein in 10 mm potassium phosphate buffer, pH 8.0, was determined in a 1-mm pathlength quartz cuvette (Starna) between 240 and 180 nm at 100 nm/min with a 1-nm pitch.

##### Isothermal Titration Calorimetry

Both the protein and ligand preparations were degassed at 2 °C below the experimental temperature immediately prior to analysis using a VP-ITC microcalorimeter (MicroCal), controlled by VPViewer2000 version 1.4.24 (MicroCal LLC). The titration pattern was an initial injection of 3 μl of the ligand, followed by 6-μl injections of 14 s and separated by 180 s. The titrations were then analyzed using Origin 7SR2 version 7.0383(B383) (OriginLab Corp.) by fitting to one set of binding sites.

##### Whole Cell [^14^C]Neu5Ac Uptake Assay

These were performed in a similar manner to Severi *et al.* (22). Briefly, overnight cultures in minimal medium with the carbon source of interest were diluted to an *A*_650_ of 0.1 in the same medium and grown to an *A*_650_ of 0.5. These cells were harvested, washed four times, and resuspended in M9 salts to an *A*_650_ of 3.0. For Neu5Ac uptake assays, cells were diluted 10-fold in M9 at 37 °C and allowed to acclimatize for 2 min with stirring, before initiating the assays by adding varying amounts of [^14^C]Neu5Ac (Sigma) appropriately diluted with unlabeled Neu5Ac. The uptake assay and total protein quantification were then performed as described in Severi *et al.* (11), except that 200 μl of cell suspensions were immobilized instead of 400 μl. *K_s_* and *V*_max_ values were calculated by fitting to a hyperbolic Michaelis-Menten equation using SigmaPlot.

##### SiaQM-Proteoliposome [^14^C]Neu5Ac Uptake Assay

*In vitro* [^14^C]Neu5Ac uptake by reconstituted SiaPQM was measured using the method of Mulligan *et al.* (12). N-terminally tagged SiaQM was purified from the *E. coli* MC1061 pBADnQM membrane fraction using nickel-nitrilotriacetic acid resin (Qiagen) and reconstituted into proteoliposomes with *E. coli* lipids by rapid dilution. Proteoliposomes containing 200 μg of SiaQM were resuspended in Inside buffer (100 mm potassium acetate, 20 mm potassium phosphate, 2 mm MgSO_4_, pH 7.0) and extruded 11 times through a 400-nm polycarbonate filter (Avestin Inc.). The extruded proteoliposomes were collected by ultracentrifugation and resuspended in 50 μl of Inside buffer. For each uptake assay, 5 μm of the binding protein of interest and 5 μm [^14^C]Neu5Ac were added to 300 μl of the reaction (Outside) buffer (100 mm sodium acetate, 2 mm MgSO_4_, 20 mm sodium PIPES, pH 7.0, 1 μm valinomycin) and incubated at the reaction temperature of 30 °C for 1 min. 6 μl of 1.15 μm SiaQM-proteoliposomes were added to start the reaction, and 50-μl samples were taken as indicated. Each of these samples was mixed with 50 μl of reaction buffer containing 1 mm unlabeled sialic acid for 10 s, added to a 0.22-μm nitrocellulose filter (Millipore), and washed with 2 ml of 50 mm potassium phosphate buffer, pH 7.0. The radioactivity associated with the filters was determined using liquid scintillation counting.

##### Bacterial Growth Experiments

*E. coli* BW25113 Δ*nanT* containing pAH15 or pES15-17 was inoculated to an *A*_650_ 0.01 in 700 μl M9 minimal medium with Neu5Ac and 1 mm IPTG in a 24-well glass-bottom plate closed by an air-permeable sterile lid. Growth at 35 °C with shaking at 250 rpm was monitored using a prototype incubated plate shaker (EnzyScreen). Shaking was halted every 30 min for about 1 min so that a flat-bed scanner could capture an image of the base of the plate. The increasing whiteness of the growing cultures was converted to a *G* value by the associated software (ImageAnalysisGIU version 1.0.0.0), which was correlated to an *A*_650_ value (*A*_650_*) using a stand curve. The density of all growth experiments was within the linear range of this.

For growth at low (1 mm) concentrations of Neu5Ac, we used a Tecan Infinite M200 Pro microplate reader. Bacteria were inoculated from a single colony into 4 ml of M9 glucose (0.4% w/v) medium and grown overnight. For strains harboring plasmids, ampicillin (100 μg/ml) and IPTG (1 mm) were added. The *A*_650 nm_ of each culture was measured and adjusted to 1 by addition of 1× M9 salts. 150 μl of M9 minimal medium with the appropriate concentration of Neu5Ac was aliquoted into the 96-well plate (Corning Costar 3595), and 1.5 μl of bacterial cell suspension was added and mixed by pipetting. For strains harboring plasmids, ampicillin (100 μg/ml) and IPTG (1 mm) were added. The outer wells of the 96-well plate were filled with 150 μl of sterile distilled H_2_O to prevent evaporation from the internal wells. The plate reader was set to monitor *A*_650 nm_ every 30 min for 48 h and to maintain a constant temperature of 37 °C. Data were exported to Magellan data analysis software for analysis. An average of two media-only control wells was taken from every 30-min time point. The data were normalized by subtracting this value from the *A*_650 nm_ reading for all cultures at the respective time point to eliminate background absorbance noise.

##### Crystallography

SiaP WT and mutant proteins at a concentration of 30–35 mg/ml were co-crystallized with the respective ligand at concentrations of 10, 15, and 20 mm (for R147K, R147A, and R147E/WT, respectively) using 300-nl sitting drops in 96-well Greiner plates composed of an equivolume of protein and reservoir buffer (100 mm MES, pH 6.0, 28.5% PEG 6K (w/v), with varying additives). Crystals obtained at 4 °C were vitrified in the presence of 10% glycerol and tested in-house before data collection at ID23-2 at the ESRF synchrotron facility in Grenoble, France, and at beamline I04 at the DIAMOND Lightsource, Didcot, UK.

Data were processed in MOSFLM and SCALA, and initial phases were obtained by rigid body refinement using 2V4C as a closed model and for the 5% *R*_free_ batch. The WT-Neu5Ac structure was solved using ACORN *ab initio* phasing to eliminate model bias at atomic resolution with a fragment subset of 800 atoms starting from atom 1 of the same model. Cycles of refinement within Refmac (version 5.5.0109) were iterated with model building in Coot (version 12). Ligand and water molecules were added in the final iterations of model building and refinement, and Babinet scaling was applied. Structures were refined anisotropically with the exception of the R147K structure (2xwi), which was refined isotropically at a resolution of 2.2 Å. Composite omit maps were calculated with Comit (23) for the placement of low occupancy water molecules that were added automatically using Coot. Finally, water molecules were checked to fulfill the following criteria: the “Density Fit Graph” and “Check Waters” validation tools in Coot, *B*-factor variance, and H-bond distances. The identity of the compounds within the WT and R147E structure was checked using unrestrained refinement on the basis of their atomic *B-*factors and bond lengths. Finally, the data were checked using Coot, Sfcheck, Procheck, and the ADIT Protein Data Bank deposition server. The superpositions and figures were generated with PyMOL version 0.99 and CorelDRAW X3, respectively.

## Results

### 

#### 

##### Arg-147 Is Essential for High Affinity Ligand Binding to SiaP in Vitro

**FIGURE 1.**
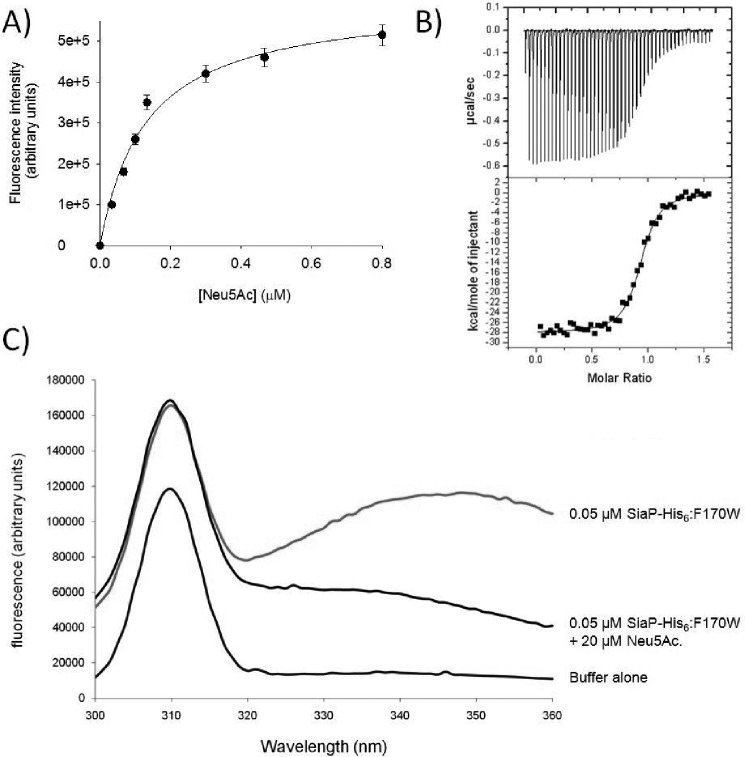
**Neu5Ac is bound by SiaP-His_6_ with high affinity.** *A,* tyrosine fluorescence changes of 0.05 μm SiaP-His_6_ upon increasing additions of Neu5Ac. *B,* fluorescence emission spectra of 0.05 μm SiaP-His_6_:F170W before and after the addition of 20 μm indicating the large quench in signal due to binding. A buffer only control is included to illustrate the scattering light from the emission signal. *C,* isothermal titration calorimetry analysis of 10 μm SiaP-His_6_ in 50 mm Tris/HCl, pH 8, at 37 °C. 150 μm Neu5Ac was injected into the cell in 6-μl aliquots.

A reanalysis of the Pfam database family for DctP-TRAP SBPs (SBP-bac-7) reveals that 5943 of 6142 full-length sequences (96.8%) have an arginine residue in an equivalent position to Arg-147 in SiaP. To investigate the involvement of SiaP residue Arg-147 in transporter function, we mutated it to either alanine (R147A) or lysine (R147K). Binding of *N*-acetylneuraminic acid (Neu5Ac) to C-terminally hexahistidine-tagged wild-type SiaP-His_6_ yielded a *K_D_* of 0.14 ± 0.04 μm, in close agreement with published data for native or N-terminally His-tagged versions (Fig. 1*A*) (8, 11). In contrast to this high nanomolar affinity, the R147A and R147K mutants analyzed in the same way gave no detectable change in fluorescence with additions of up to 3 mm Neu5Ac. To increase the optical readout signal, we generated a SiaPF170W mutant with improved optical properties, demonstrating a 50% quench in the emission signal at 340 nm upon binding of Neu5Ac (Fig. 1*B*). The effect on the binding affinity was relatively modest (*K_D_* for Neu5Ac of 1.21 ± 0.03 μm). The Arg-147 mutations were introduced into the F170W background, and we were able to detect saturable Neu5Ac binding to the F170W/ R147K mutant alone with very low affinity (*K_D_* of 38.7 ± 8.3 mm). As an alternative non-optical method, we measured binding by isothermal titration calorimetry, and although we obtained consistent data for the SiaP-His_6_ with a *K_D_* of 0.11 ± 0.02 μm (Fig. 1*C*), we could not observe binding for the mutants. The folded state of the mutant proteins was examined using CD spectra that revealed a comparable secondary structural profile to the wild-type protein suggesting that the loss of high affinity binding is not due to a loss of protein folding (data not shown).

##### High Affinity Sialic Acid Transport Is Abolished in Arg-147 Mutants

**FIGURE 2.**
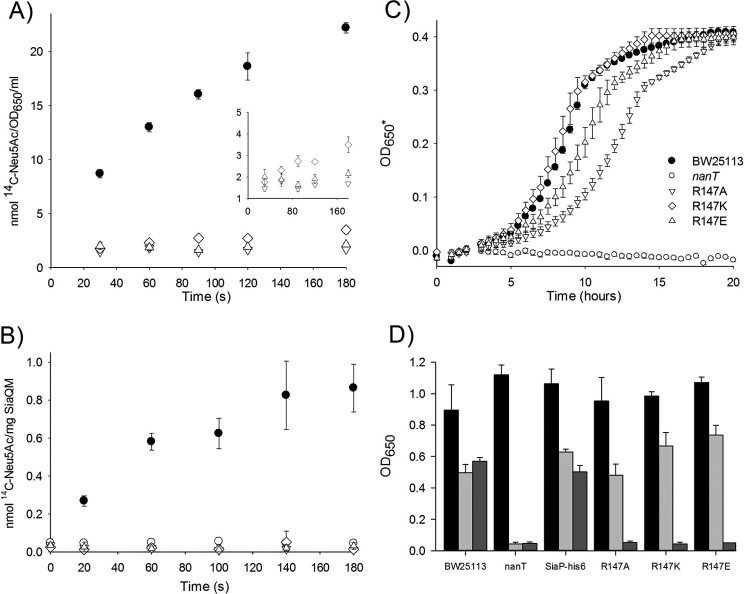
**Effects of the Arg-147 mutants on Neu5Ac transport via SiaPQM are due to reduced affinity for the ligand.** *A, in vivo* uptake of 5 μm [^14^C]Neu5Ac by *E. coli* BW25113 Δ*nanAT* mediated by IPTG-induced expression of *siaPQM* and variants from pWKS30. *Inset* shows the data for the Arg-147 mutant expanded to illustrate the detectable activity of the R147K mutant (*diamonds*). *B, in vitro* uptake of 5 μm [^14^C]Neu5Ac into SiaQM-containing proteoliposomes with a pre-generated ΔNa^+^ Δψ across the membrane mediated by 5 μm SiaP-His_6_ and variants. The negative control included used liposomes lacking SiaQM. *C,* growth of *E. coli* BW25113 Δ*nanT* in M9 minimal medium with 3.2 mm (1 mg/ml) Neu5Ac as the sole carbon source mediated by IPTG-induced expression of *siaP-His*_6_–*siaQM* and variants. Growth is reported as an apparent *A*_650_ (*A*_650_*) due to the data output of the experimental system. Positive and negative controls are shown as *filled* and *empty circles*, respectively, and the R147A, R147K, and R147E mutants are represented by *empty down triangles*, *diamonds,* and *up triangles*, respectively. All data are an average of three or four repeat experiments. *D,* final *A*_650_ values of overnight cultures supplemented with 0.4% glucose (*black*), 3.2 mm Neu5Ac (*pale gray*), and 3.2 mm KDN (*dark gray*) as the sole carbon sources.

To determine the effect of Arg-147 mutations on whole transporter function, we measured transport of Neu5Ac in both whole cell and reconstituted SiaPQM-mediated transport assays. We constructed the same *siaP* alleles (*R147A* and *R147K*) in a complementation vector that contained the complete *siaPQM* operon and was shown to restore Neu5Ac uptake to an *E. coli* Δ*nanAT* strain (22). Rapid uptake of 5 μm [^14^C]Neu5Ac into these cells was observed for the wild-type *siaP* allele (Fig. 2*A*), whereas no detectable uptake was observed with the *R147A* allele but a very low level of uptake for the *R147K* allele was detected (Fig. 2*A, inset*). We also reconstituted the membrane domains of SiaQM into proteoliposomes and measured uptake of 5 μm [^14^C]Neu5Ac with 5 μm SiaP as described by Mulligan *et al.* (12). Although we could readily measure uptake with the wild-type protein, we were unable to measure uptake with either the R147A or R147K mutants (Fig. 2*B*). Taken together, these data suggest that physiological high affinity transport by SiaPQM is abolished by the alteration of the Arg-147 residue of SiaP.

##### Arg-147/Carboxylate Interaction Is Not Essential for SBP Closure

**FIGURE 3.**
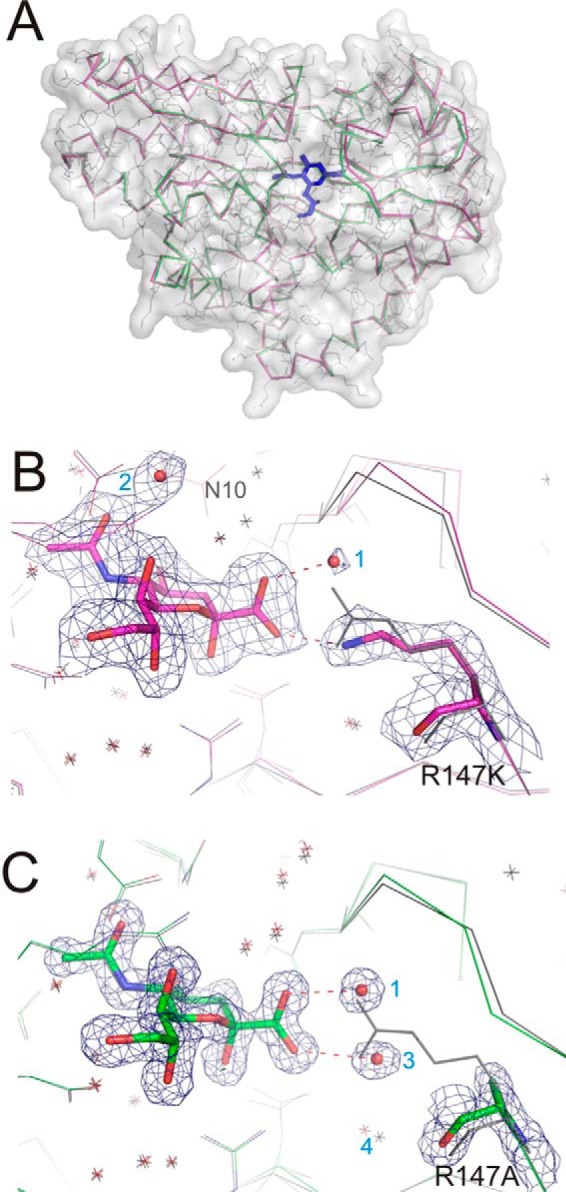
**Ligand binding induces closure of the mutants with waters substituting missing direct interactions.** *A,* structural superposition of mutants R147K (*magenta*) and R147A (*green*) onto WT (*gray*, shown with side chains) highlights that all structures exhibit a closed conformation in the presence of Neu5Ac (*blue*) and are structurally indistinguishable. *B,* close-up of the binding pocket of the R147K mutant solved to 2.2 Å resolution, with electron density (2m*F_o_* − D*F_c_* omit maps rendered at 1σ) shown, reveals that two water molecules *1* and 2 (*red spheres* labeled with *blue font*) take up interactions with opposite ends of the ligand enabled by the alternative conformation of Asn-10 and the Lys mutation. *C,* close-up of the binding pocket of R147A (2m*F_o_* − D*F_c_* omit maps rendered at 1.5σ). The Ala mutation opens space for another water (*3*) in an equivalent position to the missing guanidinium group, but without altering the surrounding backbone in comparison with the wild type (shown in *gray lines*).

The high level of conservation of the Arg-147 residue, or its equivalent in other DctP-TRAP SBPs, is unusual for a binding protein family, where sequence diversity is normally much greater. Given the almost total loss of detectable function for SiaPQM containing this single point mutant within an otherwise unaltered binding site, we wondered whether the arginine/carboxylate interaction might have additional fundamental roles in the SBP function such as domain closure or productive interactions with the SiaQM proteins. To understand the structural consequences of removing the Arg-147 from SiaP, we crystallized the R147A and R147K mutants in the presence of 10–15 mm Neu5Ac (Table 1). Surprisingly, given the very weak binding observed for these proteins *in vitro*, both structures contain Neu5Ac bound in the substrate binding pocket, and the proteins are clearly in the closed ligand-bound conformation as opposed to the expected open ligand-free conformation that has been observed for SiaP previously (Fig. 3*A*) (5, 8). Overall, both mutant structures are structurally indistinguishable from the wild-type structure in complex with Neu5Ac (3B50) (8) with a root mean square deviation (r.m.s.d.) of 0.30 and 0.17 Å for the R147K and R147A mutants, respectively. The ability of the mutant proteins to bind and close around the Neu5Ac in these conditions suggests that the interaction of Arg-147 with the carboxylate is neither essential for protein closure nor Neu5Ac accommodation. Neu5Ac is recognized in an identical fashion to the wild-type protein, albeit at a much lower affinity.

**TABLE 1:** **Data collection and refinement statistics**

SiaP	R147A	R147K	R147E	Wild type (WT)
Protein Data Bank code	2xwk	2xwi	2xwo	2xwv
Ligand	15 mm Neu5Ac	10 mm Neu5Ac	20 mm sialylamide	(20 mm “sialylamide” = ) Neu5Ac
Data collection	ID23-2 (ESRF)	ID23-2 (ESRF)	ID23-2 (ESRF)	I04 (Diamond)
Resolution range (Å)	29.02–1.49 (1.57–1.49)	43.2–2.20 (2.32–2.20)	29.06−1.54 (1.58–1.54)	43.5–1.05 (1.08–1.05)
Space group	P2_1_ 2_1_ 2_1_	P2_1_ 2_1_ 2_1_	P2_1_ 2_1_ 2_1_	P2_1_ 2_1_ 2_1_
Unit cell parameters				
*a, b, c* (Å)	47.8, 74.8, 87.1	48.5, 74.5, 86.1	47.9, 74.8, 87.2	47.7, 74.5, 86.9
α, β, γ (°)	90, 90, 90	90, 90, 90	90, 90, 90	90, 90, 90
Completeness (%)	100 (100)	99.48 (96.02)	100 (100)	97.6 (93.2)
Unique reflections	44,338 (6391)	16,495 (2347)	47,100 (6764)	141,294 (19425)
*I*/σ(*I*)	13.8 (4.2)	10.2 (2.6)	11.9 (2.5)	9.4 (2.0)
Redundancy	7.3 (7.3)	10.1 (10.3)	7.3 (7.3)	4.6 (4.4)
*R*_merge_ (%)	0.114 (0.606)	0.219 (1.111)	0.120 (0.974)	0.085 (0.668)

**Refinement and model statistics**				
*R*_work_ (%)	0.1097 (0.137)	0.198 (0.249)	0.1304 (0.207)	0.125 (0.258)
*R*_free_	0.1636 (0.226)	0.262 (0.308)	0.1789 (0.275)	0.150 (0.263)
*R*_work + test_	0.1125	0.201	0.13287	0.126
*B-*factor (Å^2^)				
All	16.8	41.7	19.5	12.7
Main chains	12.1	41.4	16.0	11.2
Side chains, solvent	20.2	42.0	22.2	14.9
Ligand	9.2	34.1	10.1	6.1
No. of atoms				
Non-H protein	2507	2512	2503	2761
Ligand	21	21	21	21
Solvent	487	209	391	
Solvent content, VS (%)	45	46	45	45
Matthew's coefficient, VM (Å^3^/Da)	2.2	2.3	2.2	2.2
r.m.s.d.				
Bond lengths (Å)	0.015	0.018	0.018	0.019
Bond angles (°)	1.45	1.15	1.51	1.74
Ramachandran plot (%)				
Preferred regions	98.58	98.21	98.58	99.04
Allowed	1.42	1.79	1.42	0.96

##### Ordered Water Molecules Mediate Ligand Contacts to the SiaP Arg-147 Mutants

We examined the structure in more detail to assess how the loss of the Arg-147 residue is still compatible with binding of Neu5Ac. With the relatively conservative substitution of arginine to an also charged lysine (Fig. 3*B*), the key guanidinium group of Arg-147 is partially substituted by the Nζ atom of Lys-147, which occupies an equivalent position (0.3 Å shift) to one of the NH_2_ groups of Arg-147 and provides one of the interactions with the ligand (Fig. 3*B*). The other contact is now mediated by a water molecule that provides the missing H-bond to the carboxylate of Neu5Ac (Fig. 3*B*). The R147A mutant protein also crystallized in a closed Neu5Ac-bound form, despite effectively reducing the highly conserved charged side chain to a methyl group (Fig. 3*C*). In contrast to the R147K mutant, the replacement of Arg-147 by alanine results in removal of all direct contacts between residue 147 and the Neu5Ac (Fig. 3*C*). Rather, two water molecules dissipate the charge of the Neu5Ac carboxylate, mediating contacts between ligand and protein. One of these water molecules (water 1) is present in both the R147K and R147A structures, although the other water (water 3) is unique to this structure.

Despite this significant change in the binding pocket, the structure of SiaP around the missing arginine is only very mildly perturbed. In both structures (2xwi and 2xwk) slightly elevated *B*-factors for the carboxylate group of Neu5Ac compared with the rest of the ligand indicate that this part of the Neu5Ac is less constrained in the absence of the strong interaction with the Arg-147 residue, compared with the wild-type protein. Water 3 also has a higher *B*-factor than water 1, which manifests as an anisotropic motion perpendicular to the carboxylate. This mobility translates to the neighboring water molecule (water 4) that moves by ∼1 Å with respect to the wild type, concomitant with a 0.5-Å translation of the β-strand that bears residue 147. There are no additional waters present in the void left by the short side chain of Ala due to the aliphatic patch behind the arginine that drives it into the binding site (4). Hence, although Arg-147 provides important contacts that are essential for high affinity binding, the required coordination of the Neu5Ac carboxylate in the binding pocket can be fulfilled by water molecules allowing these mutants to both bind and close around the Neu5Ac under these conditions.

##### High Ligand Concentrations Restore in Vivo Transporter Function for SiaP Arg-147 Mutants

**FIGURE 4.**
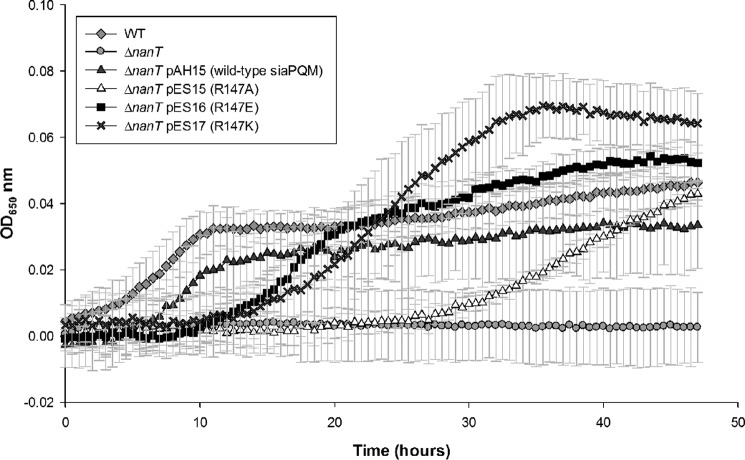
**Growth of *E. coli* strains on 1 mm Neu5Ac as the sole carbon source.** Growth of *E. coli* strains in M9 minimal medium with 1 mm Neu5Ac as the sole carbon source over 48 h with the indicated strains: wild-type (WT) (*gray diamond*); Δ*nanT* strain (*gray circle*); this same strain complemented the wild-type *siaPQM* (*dark gray triangles*) or the genes with a *siaP* mutant allele of R147A (*white triangles*), R147E (*black squares*), or R147K (*crosses*). Data are the average of at least triplicate experimental replicates.

Given that the structural data suggested that the Arg-147 mutant can close and the structure of the ligand-bound forms for the wild-type and Arg-147 mutants are indistinguishable apart from the missing Arg/carboxylate interaction, we reasoned that transporter function might be restored under similar substrate concentrations to those used for crystallography. To test this hypothesis, we used a genetic system that requires the presence of a functional sialic acid transporter to allow growth of *E. coli* in liquid media with sialic acid as the sole carbon source. In an M9 minimal medium, *E. coli* will grow on 3.2 mm (∼1 mg/ml) Neu5Ac as the sole carbon source, which is dependent on the NanT sialic acid transporter, a classical secondary transporter of the major facilitator superfamily (22, 24). A Δ*nanT* strain cannot grow on Neu5Ac unless a functional sialic acid transporter is provided in *trans,* and *siaPQM* can function in this role (22). Using the Δ*nanT* strain complemented with either the wild-type *siaPQM* or equivalent genes containing the specific Arg-147 mutants, we were indeed able to detect growth for all of the strains (Fig. 2*C*). Interestingly, we observed differential growth phenotypes for the Arg-147 mutants, with the more conservative changes in the R147K mutant exhibiting growth more similar to the strain expressing the wild-type SiaP (doubling times of ∼120 min), than the R147A mutation, which resulted in an ∼50% decrease in the growth rate, giving a doubling time of about ∼180 min. We repeated these experiments using a microplate reader with lower (1 mm) Neu5Ac concentrations and saw a similar pattern of results, although the R147A mutant growth is severely attenuated (Fig. 4). These data suggest that in the presence of relatively high external concentrations of sialic acid and in the context of transporter function in whole cells, the Arg-147 residue is not essential and in fact links the differential phenotypes for substitutions of the Arg-147 to the nature of the side-chain modifications introduced and observed in the crystal structures.

##### Weakening Protein and Ligand Interactions Simultaneously Abolish Transporter Function

*E. coli* can grow on the related nonulosonic acid KDN using *siaPQM* expressed in *trans* (25), but this compound binds to SiaP with over 300-fold lower affinity than Neu5Ac (*K_D_* of 42 μm) (5). To test whether wild-type and mutant versions of SiaPQM could function with this weaker binding substrate, we repeated the above experiments but with KDN replacing Neu5Ac as the sole carbon source. Although we could detect growth for the wild-type on KDN, there was no detectable growth of the Arg-147 mutants (Fig. 2*D*). Together these data suggest that for the physiological substrate of SiaPQM, the loss of the function of the Arg-147 can be tolerated *in vivo* at high substrate concentrations. However, for a ligand that already binds relatively weakly to the wild-type protein, the resulting drop in ligand binding affinity in the Arg-147 mutants is too great to support any physiological functioning of the transporter in the low micromolar concentrations of free Neu5Ac found in the body.

##### Engineered Arg-147 Mutant Can Accommodate Non-carboxylate-containing Ligands

The coordination of Neu5Ac in the R147A mutant suggests that waters can essentially replace the guanidinium group of the arginine and retain the ability of the protein to coordinate the carboxylate of the ligand. To investigate whether this water network adds plasticity, *i.e.* ligand promiscuity, we engineered an R147E mutant to maintain the hydrophobic part of the arginine side chain and yet have the capacity to coordinate water 1 and water 3 observed in the R147A structure. In addition, by using the charge-swap from Arg to Glu, we can also assess the ability of the water molecules to dissipate the apparent charge clash. *In vivo* data confirm the *in silico* predictions as growth of bacteria containing SiaP with the R147E mutation is more impeded than the R147K mutation but less than the R147A mutation (Fig. 2). These data suggest that the two waters are able to accommodate two carboxylate groups pointing directly toward each other, while retaining sufficient function to enable bacterial growth.

**FIGURE 5.**
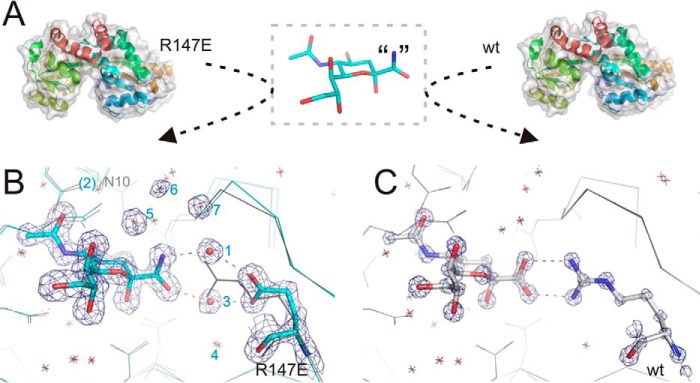
**Molecular fishing for organic acids.** *A,* both WT and R147E mutant have been exposed to a stock of non-cognate “sialylamide.” In the absence of apo-structures, 2CEY was used to depict the open form of the structure, colored from *blue* to *red* from the N to the C terminus. *B,* R147E (*cyan sticks*) exhibits a closed conformation in the presence of sialylamide, superposed onto WT (*gray lines* for side chains and *stars* for water molecules) for reference. Two waters (*red spheres, 1* and *3*) bridge the interaction between the amide and the mutated residue R147E (*red dashed lines*). Electron density (2m*F_o_* − D*F_c_* omit maps rendered at 3σ) is shown for several other waters (5–7) that are shifted in response to the amide H-bond donor. *C,* however, atomic resolution (2m*F_o_* − D*F_c_* omit maps rendered at 5σ) reveals that the wild-type protein filters out a minor impurity of sialic acid confirming that the conserved arginine confers high selectivity for organic acids; *cf.* same position of surrounding water molecules as in WT in *gray*.

To investigate further the ability of the waters to relax the selectivity of SiaP for its ligands, the R147E mutant was crystallized in the presence of the non-cognate ligand sialylamide, which differs from Neu5Ac by a change of the carboxylate to an amide (Fig. 5*A*). This neutral molecule was previously reported to bind wild-type SiaP with around a thousand-fold lower affinity than Neu5Ac (*K_D_* of 240 μm (5)). The R147E-sialylamide co-crystal structure (Fig. 5*A*) again reveals a closed conformation with an r.m.s.d. of 0.2 Å from the wild-type SiaP in complex with Neu5Ac. The amide and the opposing carboxylate group of Glu in the charge-swap mutant are linked by the two interstitial waters 1 and 3 in equivalent positions to those seen within the R147A mutant. The presence of the NH_2_ group results in the slight rearrangement of the water network around this ligand (*waters 5–7* in Fig. 5*A*) as the NH_2_ group donates rather than accepts hydrogen bonds.

We attempted to crystallize the wild-type protein with this non-cognate sialylamide ligand and were able to collect data to atomic resolution of 1.05 Å where *ab initio* phasing was applied to remove any model bias. Strikingly, the ligand appeared to be coordinated exactly as Neu5Ac, including the positioning of all waters in the binding pocket, and careful refinement of the structure revealed that the bound species was in fact Neu5Ac, based on analysis of H-bonding patterns (interaction with waters), *B*-factors of the carboxylate, and also considering expected bond lengths for unrestrained refinement of oxygen and nitrogen atoms (26). This final structure (2xwv) is indistinguishable (r.m.s.d. 0.1 Å) from the reference SiaP-Neu5Ac complex of Johnston *et al.* (8) and has the highest resolution for a TRAP SBP to date (Fig. 5). Mass spectrometry data confirmed the presence of trace amounts of Neu5Ac in the sialylamide sample despite purification after synthesis using silica and ion-exchange chromatography and checked by one-dimensional ^1^H NMR.^7^ The finding that the wild-type protein was able to selectively bind this minor contaminating component, while R147E protein exposed to the same stock crystallized with the bound sialylamide, confirms the important role of the Arg-147 residue in the selectivity of SiaP for its cognate ligand.

## Discussion

As we learn about the diversity of bacterial physiology through genome sequencing, it is clear that bacterial TRAP transporters form significant components of the encoded repertoire of solute transporter in the genomes of bacteria that live in nutrient-poor environments (7). The best examples of this are in marine environments that contain a wide range of dissolved organic compounds at low concentrations, requiring organisms that live in these environments to be able to scavenge effectively for many diverse nutrients (27). TRAP transporters appear to be enriched in these environments as they have high affinity, use Na^+^ as a coupling ion for transport, and have specificity for diverse organic acids that appear to be major carbon sources in these niches (28, 29). When TRAP transporters were first discovered, they were as part of C_4_-dicarboxylate transporters, but subsequent work has identified a range of other ligands, united by containing a negatively charged group, mostly carboxylate, or in the case of ectoine a sulfonate group (3, 7, 13, 14). More recently, around 30 novel TRAP SBPs of the DctP family had their structures solved with co-purified ligands, revealing a whole plethora of new organic acid ligands, but also examples of zwitterionic amino acids and a glycerol 3-phosphate molecule bound. In all of these structures, the carboxylate (or rarely a sulfonate or phosphate) forms an electrostatic interaction with the conserved arginine in these proteins, equivalent to Arg-147 in SiaP (18). We have now investigated using SiaP, the structural and biochemical role of this residue in TRAP transporter function.

First, the binding affinity of SiaP is significantly reduced in the absence of Arg-147. All three Arg-147 mutants have major defects in ligand binding *in vitro* and *in vivo* using physiological (micromolar) concentrations of substrate. The Arg-147 mutations do not alter the overall structure of SiaP significantly (r.m.s.d. of 0.12, 0.14, and 0.23 Å between wild-type and R147A, R147E, and R147K, respectively), and the impact of these structural changes is seen through the loss of the ionic salt bridge between the protein and the ligand. Andrews *et al.* (30) reported the average intrinsic binding energy of a carboxylate group to be 8.2 kcal/mol (in the range 7.3–10.3 kcal/mol at 298 K), and hence an important component of driving high affinity in a ligand-binding site. Given that a change in the equilibrium constant for binding (*K_d_*) by 1 order of magnitude corresponds to a change in Gibbs free energy (Δ*G*) of ∼1.36 kcal/mol, the loss of the salt bridge could lead to a reduction in *K_d_* of over 5 orders of magnitude. This corresponds to a decrease in binding affinity from 120 μm to ≥10 mm, which is very similar to our experimental observations. It supports the hypothesis that Arg-147 is essential for physiological function of TRAP transporters that bind carboxylate-containing ligands. Further support for the “affinity” hypothesis comes from experiments with KDN, a 300-fold weaker binder than Neu5Ac. KDN binding is undetectable when other parts of the interaction with the binding site are changed in addition to the broken Arg/carboxylate interaction. Hence, our data support a conserved role for this arginine in conferring high affinity to TRAP transporters mediated by an electrostatic binding interaction in the SBP subunit alone.

Our data support the essential function of Arg-147 in SiaP for the effective colonization of the host by the *H. influenzae* (9, 31). The bacterium obligately depends on scavenging sialic acid from the host via SiaPQM to incorporate it into LOS and confer serum resistance (9, 31). In this pathogen, the transporter is co-transcribed with a gene encoding a periplasmic sialic acid mutarotase (NanM, HI0148), which helps the bacterium scavenge the limited amount of α-Neu5Ac present in the environment to the β-form that is transported by SiaPQM (32). Our structural data agree with microbiological work demonstrating that LOS sialylation is lost in a bacterium containing an R147A SiaP mutation in the presence of 100 μm Neu5Ac (8).

The second significant finding from this study is that water molecules can partially replace the function of the missing guanidinium group of Arg-147. Nonetheless, faster growth with the R147K mutant over both other mutants (Fig. 2) suggests an affinity benefit from even one direct charge-mediated interaction with the ligand over losing both direct contacts compared with using two water-mediated interaction (Fig. 3, *B* and *C*). Yet this “plasticity” in the binding site can recover function at high non-physiological (millimolar) substrate concentrations. It also allowed us to engineer an R147E mutant that positions two water molecules in the space previously occupied by the guanidinium group of Arg-147. Our crystal structure confirmed that the mutant protein is able to use water-mediated contacts to bind sialylamide, an atypical ligand for SiaP. Crystallization of the wild-type SiaP with the same preparation of sialylamide revealed that the arginine is able to “fish out” even the minimal amounts of Neu5Ac present in this sialylamide preparation, analogous to conditions of limited environmental nutrients. These data also suggest that sialylamide probably is not a true ligand for wild-type SiaP, and binding detected in Muller *et al.* (5) was likely due to this low level of contamination with Neu5Ac.

In addition to the two geometrically engineered water molecules in the R147E/sialylamide structure, there is further evidence of using water to neutralize ligand charge in other SBP subtypes; the bridging of negatively charged aspartate ligands by two water molecules to the protein backbone was suggested to be a general feature of 59 out of 78 Bug proteins (33), which can be components of bacterial tripartite tricarboxylate transporters (34).

Although there is now strong support for the arginine being central to TRAP transporter ligand binding, there are small fractions of DctP-TRAP SBPs that do not have an equivalent arginine. In our Pfam analysis of the SBP-bac-7 family of 6142 sequences, 3% (199) lack the Arg-147 equivalent. The Arg is most commonly substituted for a Phe, a hydrophobic aromatic amino acid. Interestingly, the structure of the Tp0957 TRAP SBP from *Treponema pallidum* revealed a unique hydrophobic binding pocket where the Arg-147 equivalent is an alanine, which may define a small group of TRAP transporters likely binding hydrophobic substrates (35, 36), although the exact nature of these ligands is currently unknown. Within the study by Vetting *et al.* (18), the structure of *Chromohalobacter salexigens* DSM 3043 Csal_0678 protein, which lacks the Arg-147 equivalent, binds an atypical TRAP ligand, ethanolamine, reinforcing the strong link between the Arg and selectivity of carboxylate-containing ligands as typical TRAP substrates.

In conclusion, our data provide the first structural, biochemical, and mechanistic insight into the importance of Arg-147 in TRAP transporter biology. Its function lies in conferring high ligand specificity and affinity to the TRAP transporters and enables function in environments with limited amounts of carboxylate-containing ligands.

## Author Contributions

G. H. T. conceived and coordinated the study. A. P. H., M. F., E. S., and D. B. designed, performed, and analyzed the experiments. J. H. was involved in doing the experiments. A. G. W. and R. E. H. provided assistance to data interpretation. G. H. T., A. P. H., and M. F. wrote the paper. All authors reviewed the results and approved the final version of the manuscript.

## Footnotes

7 A. Watts, unpublished data.

## Footnotes

6 The abbreviations used are:

TRAP: tripartite ATP-independent periplasmic
SBP: substrate-binding protein
r.m.s.: root mean-squared
Neu5Ac: *N*-acetylneuraminic acid
TMH: transmembrane helix
IPTG: isopropyl 1-thio-β-d-galactopyranoside
LOS: lipooligosaccharide.

## References

[1] Ren Q., Chen K., and Paulsen I. T. (2007) TransportDB: a comprehensive database resource for cytoplasmic membrane transport systems and outer membrane channels. Nucleic Acids Res. 35, D274–D279 17135193 10.1093/nar/gkl925 PMC1747178

[2] Paulsen I. T., Nguyen L., Sliwinski M. K., Rabus R., and Saier M. H. Jr. (2000) Microbial genome analyses: comparative transport capabilities in 18 prokaryotes. J. Mol. Biol. 301, 75–100 10926494 10.1006/jmbi.2000.3961

[3] Forward J. A., Behrendt M. C., Wyborn N. R., Cross R., and Kelly D. J. (1997) TRAP transporters: a new family of periplasmic solute transport systems encoded by the dctPQM genes of *Rhodobacter capsulatus* and by homologs in diverse Gram-negative bacteria. J. Bacteriol. 179, 5482–5493 9287004 10.1128/jb.179.17.5482-5493.1997 PMC179420

[4] Fischer M., Zhang Q. Y., Hubbard R. E., and Thomas G. H. (2010) Caught in a TRAP: substrate-binding proteins in secondary transport. Trends Microbiol. 18, 471–478 20656493 10.1016/j.tim.2010.06.009

[5] Müller A., Severi E., Mulligan C., Watts A. G., Kelly D. J., Wilson K. S., Wilkinson A. J., and Thomas G. H. (2006) Conservation of structure and mechanism in primary and secondary transporters exemplified by SiaP, a sialic acid binding virulence factor from *Haemophilus influenzae*. J. Biol. Chem. 281, 22212–22222 16702222 10.1074/jbc.M603463200

[6] Davidson A. L., Dassa E., Orelle C., and Chen J. (2008) Structure, function, and evolution of bacterial ATP-binding cassette systems. Microbiol. Mol. Biol. Rev. 72, 317–364 18535149 10.1128/MMBR.00031-07 PMC2415747

[7] Mulligan C., Fischer M., and Thomas G. H. (2011) Tripartite ATP-independent periplasmic (TRAP) transporters in bacteria and archaea. FEMS Microbiol. Rev. 35, 68–86 20584082 10.1111/j.1574-6976.2010.00236.x

[8] Johnston J. W., Coussens N. P., Allen S., Houtman J. C., Turner K. H., Zaleski A., Ramaswamy S., Gibson B. W., and Apicella M. A. (2008) Characterization of the *N*-acetyl-5-neuraminic acid-binding site of the extracytoplasmic solute receptor (SiaP) of nontypable *Haemophilus influenzae* strain 2019. J. Biol. Chem. 283, 855–865 17947229 10.1074/jbc.M706603200

[9] Jenkins G. A., Figueira M., Kumar G. A., Sweetman W. A., Makepeace K., Pelton S. I., Moxon R., and Hood D. W. (2010) Sialic acid-mediated transcriptional modulation of a highly conserved sialometabolism gene cluster in *Haemophilus influenzae* and its effect on virulence. BMC Microbiol. 10, 48 20158882 10.1186/1471-2180-10-48 PMC2836998

[10] Allen S., Zaleski A., Johnston J. W., Gibson B. W., and Apicella M. A. (2005) Novel sialic acid transporter of *Haemophilus influenzae*. Infect. Immun. 73, 5291–5300 16113244 10.1128/IAI.73.9.5291-5300.2005 PMC1231074

[11] Severi E., Randle G., Kivlin P., Whitfield K., Young R., Moxon R., Kelly D., Hood D., and Thomas G. H. (2005) Sialic acid transport in *Haemophilus influenzae* is essential for lipopolysaccharide sialylation and serum resistance and is dependent on a novel tripartite ATP-independent periplasmic transporter. Mol. Microbiol. 58, 1173–1185 16262798 10.1111/j.1365-2958.2005.04901.x

[12] Mulligan C., Geertsma E. R., Severi E., Kelly D. J., Poolman B., and Thomas G. H. (2009) The substrate-binding protein imposes directionality on an electrochemical sodium gradient-driven TRAP transporter. Proc. Natl. Acad. Sci. U.S.A. 106, 1778–1783 19179287 10.1073/pnas.0809979106 PMC2644114

[13] Kuhlmann S. I., Terwisscha van Scheltinga A. C., Bienert R., Kunte H. J., and Ziegler C. (2008) 1.55 A structure of the ectoine-binding protein TeaA of the osmoregulated TRAP-transporter TeaABC from *Halomonas elongata*. Biochemistry 47, 9475–9485 18702523 10.1021/bi8006719

[14] Lecher J., Pittelkow M., Zobel S., Bursy J., Bönig T., Smits S. H., Schmitt L., and Bremer E. (2009) The crystal structure of UehA in complex with ectoine-A comparison with other TRAP-T binding proteins. J. Mol. Biol. 389, 58–73 19362561 10.1016/j.jmb.2009.03.077

[15] Rucktooa P., Antoine R., Herrou J., Huvent I., Locht C., Jacob-Dubuisson F., Villeret V., and Bompard C. (2007) Crystal structures of two *Bordetella pertussis* periplasmic receptors contribute to defining a novel pyroglutamic acid binding DctP subfamily. J. Mol. Biol. 370, 93–106 17499270 10.1016/j.jmb.2007.04.047

[16] Gonin S., Arnoux P., Pierru B., Lavergne J., Alonso B., Sabaty M., and Pignol D. (2007) Crystal structures of an extracytoplasmic solute receptor from a TRAP transporter in its open and closed forms reveal a helix-swapped dimer requiring a cation for α-keto acid binding. BMC Struct. Biol. 7, 11 17362499 10.1186/1472-6807-7-11 PMC1839085

[17] Akiyama N., Takeda K., and Miki K. (2009) Crystal structure of a periplasmic substrate-binding protein in complex with calcium lactate. J. Mol. Biol. 392, 559–565 19631222 10.1016/j.jmb.2009.07.043

[18] Vetting M. W., Al-Obaidi N., Zhao S., San Francisco B., Kim J., Wichelecki D. J., Bouvier J. T., Solbiati J. O., Vu H., Zhang X., Rodionov D. A., Love J. D., Hillerich B. S., Seidel R. D., Quinn R. J., et al. (2015) Experimental strategies for functional annotation and metabolism discovery: targeted screening of solute binding proteins and unbiased panning of metabolomes. Biochemistry 54, 909–931 25540822 10.1021/bi501388y PMC4310620

[19] Neidhardt F. C., Bloch P. L., and Smith D. F. (1974) Culture medium for enterobacteria. J. Bacteriol. 119, 736–747 4604283 10.1128/jb.119.3.736-747.1974 PMC245675

[20] Zheng L., Baumann U., and Reymond J. L. (2004) An efficient one-step site-directed and site-saturation mutagenesis protocol. Nucleic Acids Res. 32, e115 15304544 10.1093/nar/gnh110 PMC514394

[21] Wang R. F., and Kushner S. R. (1991) Construction of versatile low-copy-number vectors for cloning, sequencing and gene expression in *Escherichia coli*. Gene 100, 195–199 2055470

[22] Severi E., Hosie A. H., Hawkhead J. A., and Thomas G. H. (2010) Characterization of a novel sialic acid transporter of the sodium solute symporter (SSS) family and *in vivo* comparison with known bacterial sialic acid transporters. FEMS Microbiol. Lett. 304, 47–54 20100283 10.1111/j.1574-6968.2009.01881.x

[23] Winn M. D., Ballard C. C., Cowtan K. D., Dodson E. J., Emsley P., Evans P. R., Keegan R. M., Krissinel E. B., Leslie A. G., McCoy A., McNicholas S. J., Murshudov G. N., Pannu N. S., Potterton E. A., Powell H. R., et al. (2011) Overview of the CCP4 suite and current developments. Acta Crystallogr. D Biol. Crystallogr. 67, 235–242 21460441 10.1107/S0907444910045749 PMC3069738

[24] Vimr E. R., and Troy F. A. (1985) Identification of an inducible catabolic system for sialic acids (nan) in *Escherichia coli*. J Bacteriol. 164, 845–853 3902799 10.1128/jb.164.2.845-853.1985 PMC214328

[25] Hopkins A. P., Hawkhead J. A., and Thomas G. H. (2013) Transport and catabolism of the sialic acids *N*-glycolylneuraminic acid and 3-keto-3-deoxy-d-glycero-d-galactonononic acid by *Escherichia coli* K-12. FEMS Microbiol. Lett. 347, 14–22 23848303 10.1111/1574-6968.12213

[26] Engh R. A., and Huber R. (1991) Accurate bond and angle parameters for x-ray protein structure refinement. Acta Crystallogr. A 47, 392–400

[27] Williams T. J., and Cavicchioli R. (2014) Marine metaproteomics: deciphering the microbial metabolic food web. Trends Microbiol. 22, 248–260 24731505 10.1016/j.tim.2014.03.004

[28] Poretsky R. S., Sun S., Mou X., and Moran M. A. (2010) Transporter genes expressed by coastal bacterioplankton in response to dissolved organic carbon. Environ. Microbiol. 12, 616–627 19930445 10.1111/j.1462-2920.2009.02102.x PMC2847192

[29] Sowell S. M., Abraham P. E., Shah M., Verberkmoes N. C., Smith D. P., Barofsky D. F., and Giovannoni S. J. (2011) Environmental proteomics of microbial plankton in a highly productive coastal upwelling system. ISME J. 5, 856–865 21068774 10.1038/ismej.2010.168 PMC3105774

[30] Andrews P. R., Craik D. J., and Martin J. L. (1984) Functional group contributions to drug-receptor interactions. J. Med. Chem. 27, 1648–1657 6094812 10.1021/jm00378a021

[31] Bouchet V., Hood D. W., Li J., Brisson J. R., Randle G. A., Martin A., Li Z., Goldstein R., Schweda E. K., Pelton S. I., Richards J. C., and Moxon E. R. (2003) Host-derived sialic acid is incorporated into *Haemophilus influenzae* lipopolysaccharide and is a major virulence factor in experimental otitis media. Proc. Natl. Acad. Sci. U.S.A. 100, 8898–8903 12855765 10.1073/pnas.1432026100 PMC166410

[32] Severi E., Müller A., Potts J. R., Leech A., Williamson D., Wilson K. S., and Thomas G. H. (2008) Sialic acid mutarotation is catalyzed by the *Escherichia coli* β-propeller protein YjhT. J. Biol. Chem. 283, 4841–4849 18063573 10.1074/jbc.M707822200

[33] Herrou J., Bompard C., Antoine R., Leroy A., Rucktooa P., Hot D., Huvent I., Locht C., Villeret V., and Jacob-Dubuisson F. (2007) Structure-based mechanism of ligand binding for periplasmic solute-binding protein of the Bug family. J. Mol. Biol. 373, 954–964 17870093 10.1016/j.jmb.2007.08.006

[34] Winnen B., Hvorup R. N., and Saier M. H. Jr. (2003) The tripartite tricarboxylate transporter (TTT) family. Res. Microbiol. 154, 457–465 14499931 10.1016/S0923-2508(03)00126-8

[35] Deka R. K., Brautigam C. A., Goldberg M., Schuck P., Tomchick D. R., and Norgard M. V. (2012) Structural, bioinformatic, and *in vivo* analyses of two *Treponema palladium* lipoproteins reveal a unique TRAP transporter. J. Mol. Biol. 416, 678–696 22306465 10.1016/j.jmb.2012.01.015 PMC3289903

[36] Brautigam C. A., Deka R. K., Schuck P., Tomchick D. R., and Norgard M. V. (2012) Structural and thermodynamic characterization of the interaction between two periplasmic *Treponema palladium* lipoproteins that are components of a TPR-protein-associated TRAP transporter (TPAT). J. Mol. Biol. 420, 70–86 22504226 10.1016/j.jmb.2012.04.001 PMC3367087

